# Microbial Purification of Postfermentation Medium after 1,3-PD Production from Raw Glycerol

**DOI:** 10.1155/2013/949107

**Published:** 2013-10-02

**Authors:** Daria Szymanowska-Powałowska, Joanna Piątkowska, Katarzyna Leja

**Affiliations:** Department of Biotechnology and Food Microbiology, Poznań University of Life Sciences, ul. Wojska Polskiego 48, 60-527 Poznan, Poland

## Abstract

1,3-Propanediol (1,3-PD) is an important chemical product which can be used to produce polyesters, polyether, and polyurethanes. In the process of conversion of glycerol to 1,3-PD by *Clostridium* large number of byproducts (butyric, acetic and lactic acid) are generated in the fermentation medium. The aim of this work was to isolate bacteria strains capable of the utilization of these byproducts. Screening of 30 bacterial strains was performed using organic acids as carbon source. Selected isolates were taxonomically characterized and identified as *Alcaligenes faecalis* and *Bacillus licheniformis*. The most active strains, *Alcaligenes faecalis* JP1 and *Bacillus licheniformis* JP19, were able to utilize organic acids almost totally. Finally, it was find out that by the use of coculture (*C. butyricum* DSP1 and *A. faecalis* JP1) increased volumetric productivity of 1,3-PD production (1.07 g/L/h) and the yield equal to 0.53 g/g were obtained in bioreactor fermentation. Moreover, the only by-product present was butyric acid in a concentration below 1 g/L.

## 1. Introduction

As the production of biofuels from raw materials continuously increases, optimization of production processes is necessary. A very important issue is the development of wasteless methods of biodiesel production. One way of utilization of glycerol generated in biodiesel production is its microbial conversion to 1,3-PD [1]. However, during this process different accompanying metabolites, such as organic acids and ethanol, are also synthesized from which the main product, 1,3-PD, must be separated [2, 3]. Difficulties in its separation arise from its high boiling point and the presence of two hydroxyl groups which make it strongly hydrophilic, and therefore complicate its extraction. There are many methods of purification of 1,3-PD [4, 5]; however, physical and mechanical methods of purification are very expensive. An effective method of 1,3-PD separation is an evaporation process coupled to vacuum distillation. However, the main disadvantages of that method are high demand for energy, and, moreover, the need to remove proteins and salts before this process. Other methods of purification also have limitations. Electrodialysis, used for desalination before evaporation, causes low product yield due to losses. Liquid-liquid extraction requires large amounts of solvent. Cyclic sorption and desorption on zeolite also poses problems, such as the requirement of dewatering step and high chance of contamination (due to uninterrupted link between the bioreactor and the separation equipment). Reactive extraction is a relatively complicated process. The removal of proteins, ethanol, and salts is necessary before the reaction. Additionally, trace amounts of aldehyde in 1,3-PD interferes with the polymerization of PTT. Aqueous two-phase extraction requires large amounts of methanol and difficulty in separation of the two alcohols occur. Chromatography consumes more energy than the simple evaporation and distillation, and while high overall purity and yield of 1,3-PD can be obtained, the resulting 1,3-PD solution is not concentrated but diluted because of the low selectivity and capacity of resin or absorbent. Additionally, the efficiency of chromatographic matrices is susceptible to deterioration if feeds are not desalinated and deproteinized [4].

 Alternative purification solutions are under investigation, among which microbiological ways of utilization of by-products are very interesting and promising [6–8]. Many reports exist concerning the use of coculture in 1,3-PD production [9–11]. There is, however, a lack of information on cocultures formed by a 1,3-PD producer and a microorganism capable of utilization of 1,3-PD by-products. Such a solution could result in better overall process productivity and facilitate the downstream processing.

 The aim of this work was the isolation of new strains able to utilize organic acids, as sole carbon sources, which are generated during the production of 1,3-PD from glycerol. Furthermore, the possibility of enhancing the kinetic parameters of microbial 1,3-PD productions by the use of coculture were investigated.

## 2. Materials and Methods

### 2.1. Pure Culture Inoculum

In the conversion process of raw glycerol to 1,3-PD a bacteria strain, *C. butyricum* DSP1, was used. *C. butyricum *DSP1 was previously isolated from ruminal fluid and collected in the Department of Biotechnology and Food Microbiology, Poznan University of Life Sciences Poland, and deposited at the Polish Collection of Microorganisms PCM.

The strain was maintained in Reinforced Clostridial Medium (RCM, Oxoid, UK) in serum bottles at 4°C. Precultures of pure culture inoculum were cultivated in Hungate test tubes in appropriate cultivation media (37°C, 18 h). Bacteria from the genus *Clostridium *were cultured in a chamber for cultivation of anaerobic microorganisms (the Whitley MG500, Don Whitley Scientific, Shipley, UK), without pH regulation and stirring.

The enrichment of isolated bacteria *A. faecalis* biomass was carried out in medium (CM) consisting of (per liter deionized water): 2.0 g C_2_H_7_NO_2_, 2.0 g K_2_HPO_4_, 0.2 g MgSO_4_ and 50 *μ*L solution of CaCl_2_. Precultures of pure culture inoculum were incubated under relative anaerobic conditions in an incubation chamber (32°C, 20 h).

### 2.2. Isolation and Screening Medium

The isolation medium for new strains was Nutrient Broth Agar and glucose (2%). The composition of the screening medium was (per liter deionized water): 33.5 g C_3_H_8_O_2_, 4.65 g C_3_H_7_COOH, 1.69 g CH_3_COOH, 2.76 g C_2_H_4_OHCOOH, 3.0 g C_3_H_5_(OH)_3_, and 0.34 g C_2_H_5_OH.

### 2.3. Isolation Process

Bacteria strains able to organic acids utilization were isolated from typical sources of methanogenic fermentations (fermented chocolate, ensilages, and slurry). The first stage in the isolation of the desired strains was based on the pour-plate method. After the incubation period (48 h, 32°C), single colonies that had different morphological traits and the characteristics of a given bacterium genus were collected using disposable loops. Cells were maintained at −80°C in the culture broth supplemented with 20% glycerol.

### 2.4. Screening

Obtained isolates were cultivated on postfermentation broth. After the production of 1,3-PD from glycerol and biomass separation, permeate was inoculated by *A. faecalis* and *B. licheniformis* bacteria isolates (10% v/v). The process was carried out for 7 days, in 32°C, without pH regulation (pH at the beginning of the process adjusted to 7.15), and in strictly anaerobic conditions. Samples were taken in 24 hours intervals—the changes of glycerol, 1,3-PD, and organic acids were analyzed by HPLC. 

### 2.5. Bacterial Identification

Total DNA from bacteria was extracted with Genomic Mini AX Bacteria Kit (A & A Biotechnology, Gdansk, Poland) after an initial 1 h incubation in 50.0 mg/mL lysozyme (Sigma, Poland) at 37°C. Sequences encoding small subunits of 16S rRNA were amplified by PCR using SDBact0008aS20 and SUniv1492bA21 primers [12]. The PCR products were purified using the Clean-up Kit (A & A Biotechnology, Gdansk, Poland) and sequenced at Genomed (Warsaw, Poland) using the primers used for PCR and a primer for an inner sequence (GTGCCAGCMGCCGCCCTAA). The sequences obtained were arranged into contigs and identified using the BLAST service of the GenBank database [13].

### 2.6. Phylogenetic Analyses

The sequences encoding the 16S rRNA of *C. butyricum* DSP1 and *A. faecalis* strains were compared with the randomly selected sequences of *Clostridium *sp. and *Alcaligenes *sp. in GenBank. The sequences were aligned using the ClustalW program as implemented in BioEdit (version 7.0.9). The phylogenetic analyses were conducted using the MEGA 4.0 software [12]. The neighbour-joining method was used for phylogenetic reconstruction, and the p-distance was used for distance analysis. The best phylogenetic distance tree is shown.

### 2.7. Fermentation Medium

The composition of the fermentation medium was (per liter deionized water): 0.26 g K_2_HPO_4_; 0.02 g KH_2_PO_4_; 1.23 g (NH_4_)_2_SO_4_; 0.1 g MgSO_4_ × 7H_2_O; 0.01 g CaCl_2_ × 2H_2_O; 0.01 g FeCl_2_ × 7H_2_O, and 2.0 g yeast extract. The fermentation medium was supplemented with crude glycerol (Wratislavia-Bio, Wroclaw, Poland) at a concentration of 80.0 ± 1.0 g/L in batch fermentation. The crude glycerol composition was (w/w) 85.6% glycerol, 6% NaCl, 11.2% moisture, and pH 6.5. The media were autoclaved (121°C, 20 min.).

### 2.8. Fermentation Experiments

Fermentations were carried out in bioreactor (2L) (Sartorius Stedim, Germany). The temperature of the process was 37°C, stirring rate was 60 rpm, pH was automatically regulated with 5 M NaOH at 7.0 ± 0.1. The process was carried out using two bacteria strains. At the beginning of the process, bacteria were grown separately in two vessels (200 mL) connected to the bioreactor. In the first vessel *C. butyricum *DSP1 was cultivated on RCM medium, and in the second, *A. faecalis *JP1 was cultivated on CM medium. This preliminary stage was continued for 18–20 hours. After this time, the medium in the bioreactor was inoculated with *C. butyricum* DSP1 (using peristaltic pump) and the synthesis of 1,3-PD started. When the concentration of metabolites (both 1,3-PD and organic acids) increased, the second bacteria culture—*A. feacalis *JP1—was fed into the bioreactor. Samples from the bioreactor were taken at intervals every 2–5 hours. All data presented are means of two independent experiments performed under the same culture conditions. 

### 2.9. Analytical Methods

1,3-PD, glycerol, and organic acids were assayed by high performance liquid chromatography. 

Samples for chemical analysis were first centrifuged at 10,000 g for 10 min at 4°C (Multifuge 3SR, Germany), filtered through a 0.22 *μ*m membrane filter (Millex-GS, Millipore, USA), and then analyzed on an HPLC system (Agilent Technologies 1200 series).

Agilent Technolgies 1200 series system equipped with a refractive index detector was used. Analyses were performed isocratically at a flow rate of 0.6 mL/min. on an Aminex HPX-87H 300 × 7.8 column (Bio-Rad, CA, USA) at a constant temperature of 65°C. H_2_SO_4_ (0.5 mN) was the mobile phase. External standards were applied for identification and quantification of peaks area. Retention times (Rt) determined for the targeted compounds for were as follows: 1,3-PD—17.17 min; glycerol—13.03 min; butyric acid—20.57 min; acetic acid—14.4 min; lactic acid—11.19 min; ethanol—21.34 min.

#### 2.9.1. Determination of Interactions between of *A. feacalis* Jp1, *B. licheniformis* Jp19 and *C. butyricum* Dsp1

In order to verify if any antagonisms exist between the tested *A. feacalis* JP1 and *B. licheniformis *JP19 strains in relation to *C. butyricum* DSP1 analyses were conducted, including preparation of culture media for *A. feacalis* JP1 and *B. licheniformis* JP19 strains, separation of culture media into fractions (the supernatant and precipitate), preparation of *C. butyricum* DSP1 and analyses of activity of the obtained liquid culture and the supernatant by the well method.

#### 2.9.2. Preparation of Culture Liquid Media

Antibacterial activity was determined using 24 h cultures of *A. feacalis* JP1 (media CA) and *B. licheniformis *JP19 (media with 2% glucose). Cultures were run at a temperature of 32°C. 

#### 2.9.3. Separation of the Culture Liquid Media into Fractions

In order to obtain supernatants (S), the cultures of analyzed strains were centrifuged (5000 ×g; 10 min). Analyses of the supernatant were aimed at the determination of antagonistic activity of bacterial exocellular metabolites.

#### 2.9.4. Preparation of Indicator Microorganisms

Indicator microorganism (*C. butyricum* DSP1) was transferred to test tubes containing 10 mL RCM medium (to proliferate biomass). Cultures were run at 37°C for 24 h. Then, in order to obtain a distinct confluent layer, the liquefied agar medium was inoculated with 10% (v/v) 24 h culture of the indicator culture and poured onto Petri dishes. 

#### 2.9.5. Analyses of Antibacterial Activity of Liquid Culture Medium and the Supernatant Fraction

After solidification of the Tryptose Sulfite Cycloserine Agar (TSC, Oxoid, UK) inoculated with indicator microorganism, wells were made using a cork borer. Each well was supplemented with 150 *μ*L liquid culture medium or 150 *μ*L supernatant fraction of the analyzed strain. After incubation (24 h, 32°C, anaerobic), the diameters of the zones of growth inhibition were measured. Bacteriostatic properties were determined by measuring the growth inhibition zone diameter (growth limitation of the indicator strain).

## 3. Results

### 3.1. Isolation and Characterization of Microorganisms Able to Organic Acids Utilizations

There is only little literature data about purification of postfermentation broths in the microbial 1,3-PD synthesis [10]. Chemical purification processes are expensive [4]. Thus, in this work, the perspectives of utilization of microorganisms to remove the by-products, synthesized during the production of 1,3-PD from glycerol was considered. As an isolation source probes from methanogenic fermentation were chosen. Finally, 30 bacteria isolates were obtained. These were cultured on postfermentation broth of typical composition for propanediol fermentation carried out by *C. butyricum*. The aim was to screen for microorganisms able to decrease the concentrations of organic acids and ethanol without changing the amount of 1,3-PD. Changes in concentrations of compounds in the postfermentation broth after 7 days of cultivation are presented in Table 1.

Fifteen strains exerting the most significant influence on the broth composition (Table 1) were selected for further study. Unfortunately, some strains utilized both—organic acids and 1,3-PD. It disqualified these strains for the purpose of postfermentation broth purification. Also strains utilizing glycerol were omitted. 

Nine of the obtained strains had significant influence of the amount of organic acids and had no influence on 1,3-PD level. Strains number 1, 12, 15, and 19 were able to partially or completely utilize organic acids. The best isolates were selected for identification. Five of them belonged to the *Alcaligenes faecalis* species and four to *Bacillus licheniformis* (Table 2). Some strains (of the 15 selected isolates) were able to utilize organic acids very slowly. For example, strain no 1 metabolized it in 24 hours, while strain no 15 in 120 hours. For this reason, *A. faecalis* JP1 (isolate no 1) was chosen for further experiments.

**Figure 1:**
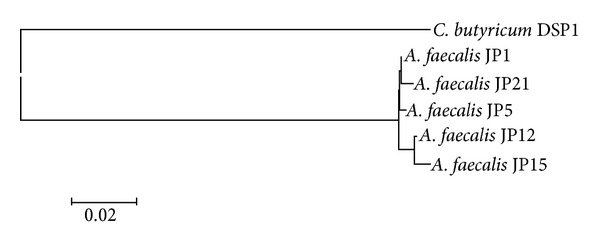
16S rRNA-based phylogenetic tree showing the position *C. butyricum* DSP1 among related *A. faecalis* strains.

To determine the relationship between the *C. butyricum* DSP1 strain and the *A. faecalis* strains, a phylogenetic tree was built based on the nucleotide sequences of the genes encoding 16S rRNA (Figure 1).

The antibacterial activity test indicated that strain *A. faecalis *JP1 has no antagonistic activity towards *C. butyricum* DSP1 used in 1,3-PD production. However, strains *B. licheniformis* inhibits growth of *C. butyricum *strain (data not shown). 

### 3.2. Cultivation of Coculture Consisting of *C. butyricum* Dsp1 and *A. faecalis* Jp1

**Figure 2:**
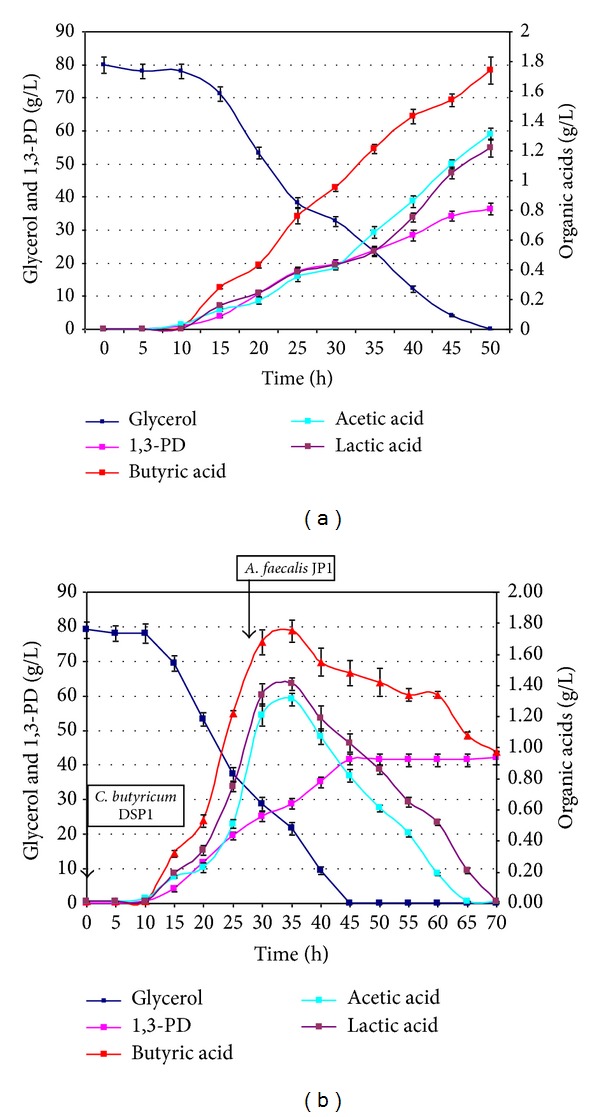
Changes in substrate and metabolites concentration during the conversion of glycerol to 1,3-PD by (a) monoculture of *C. butyricum* DSP1 and (b) coculture of *C. butyricum* DSP1 and *A. faecalis* JP1.

Fermentation by-products cause toxic stress which can damage bacteria cells, presence of these products also inhibits polymerization reactions in polyurethane production [14, 15]. Thus, efforts were taken to remove the by-products of the glycerol conversion to 1,3-PD in a microbiological way. Two bacteria strains were used *C. butyricum* DSP1 and *A. faecalis* JP1. In the first step of the experiment the fermentation medium was inoculated with *C. butyricum*. The synthesis of 1,3-PD started ca. 13 hour after inoculation. Glycerol was utilized and 1,3-PD was synthesized. During the fermentation, as the amount of 1,3-PD reached 25 g/L, butyric acid level was at 1.68 g/L, lactic acid—1.34 g/L, and acetic acid—1.21 g/L, an inoculum culture of *A. faecalis* was added to the fermentation tank. During the first 3 h after inoculation with the second strain, the level of 1,3-PD was still increasing. In 32 h of fermentation, a significant decrease (up to 20%) of lactic and acetic acid concentration was observed. Simultaneously, the productivity of 1,3-PD increased (between 31 and 35 hour of fermentation it was equal 1.55 g/L/h). Complete glycerol utilization was observed in 39 h, the yield of 1,3-PD production was 0.53 g/g crude glycerol. The process was carried until complete lactic and acetic acids utilization (Figure 2(b)).

## 4. Discussion

Methanogenic fermentation is a very interesting source of microorganism which can be applied in industrial processes of glycerol utilization [10, 11, 16]. The reason for this situation is probably the fact that glycerol is often added into methanogenic fermentation as an additional carbon source [17]. Thus, microorganisms obtained from this source tolerate high osmotic pressures. In this work, strains from silage fermented with manure and fermented chocolate were obtained. During methane production (in the acido- and acetogenesis steps) some organic acids are produced. Next, these acids are converted to gases. Thus, the authors selected such isolation sources to obtain strains resistant to high concentration of organic acids and able to utilize it. Finally, 30 isolates were obtained and their metabolic activity was tested. The best strains were *A. faecalis* JP1 and *B. licheniformis *JP19. Organic acids such as acetic acid and butyric acid are formed as by-products in the fermentation of glycerol to 1,3-PD. These compounds inhibit the growth of *C. butyricum* and deteriorate its ability to biotransform glycerol to 1,3-PD. The acids formed increase the hydrogen ion concentration in the fermentation broth, diffuse into the cell, and can lead to its death. In order to prevent it, the activity of proton pumping proteins increases resulting in higher ATP consumption. The activation of this defense mechanism leads to decreased cell metabolic activity, lower production of biomass, and metabolites. Biebl [18] described the influence of butyric and acetic acids (apart from 1,3-PD, the main primary metabolites of propanediol fermentation) on *C. butyricum*. The author determined the concentrations that inhibited the growth of the bacterium: butyric acid—19 g/L and acetic acid—27 g/L. Thus, the limitation by organic acid concentration during 1,3-PD synthesis is a very important issue, especially in fed-batch fermentations and at the end of the process. Removal of these by-products can increase the metabolic activity of bacteria producing 1,3-PD. Colin et al. [19] examined the influence of the addition of butyrate and acetate on *C. butyricum* CNCM 1211 strain during conversion of glycerol into 1,3-PD. The addition of these metabolites in concentrations of 2.5–15.0 g/L distinctly affected the bacterium viability and its metabolism. Significant observations were made during the research. The addition of acetate triggered an increase in biomass concentration and butyrate production, and at the same time reduced the yield of 1,3-PD, whereas the addition of butyrate resulted in increased diol synthesis, reduction in biomass, and butyrate production. The presented results enable to propose a thesis that reduction in butyrate synthesis in the cell ensures the appropriate amount of NADH, which is vital for the synthesis of 1,3-propanediol. In the present study, partial removal of butyric acid did not influence the number of microorganisms because the biomass already reached the plateau before inoculation with *A. faecalis* JP1. The decreased amounts of acetic and lactic acids obtained in cocultures were accompanied by better fermentation parameters then the control. The obtained yield and volumetric productivities were 15% and 30% higher, respectively. Other reports exist that concern the use of cocultures for enhanced 1,3-PD production [8, 9]. Selembo et al. [8] performed a model experiment with *C. butyricum* and *Methanosarcina mazei, *a microorganism capable of utilization of the 1,3-PD fermentation by-products. Bizukojc et al. [9] used an unidentified coculture to convert glicerol to 1,3-PD and hydrogen. Hereby, selected strain of the *Alcaligenes* genera possesses some very beneficial properties that make it fit for cocultures with *C. butyricum*. The optimal temperature for the *Alcaligenes* is in the range of 30–37°C, which complies with optimal temperature for the *Clostridium*. Bacteria form the *Alcaligenes* genera shows the ability to consume oxygen and generate anaerobic conditions necessary for *C. butyricum* strain. It seems that both bacteria can live in syntrophy. On the other hand, because of the determined antagonism, the isolated *B. licheniformis *JP19 strain may be used for the purification of 1,3-PD but not in direct coculture with *C. butyricum.* The antagonistic effects result probably from the production of proteolytic and bacteriolytic enzymes (such as n-acetylmuramoyl-l-alanine amidase, EC 3.5.1.2.8) [20]. As *B. licheniformis* has tolerance to high concentration of organic acids and solvents it could still be utilized after a separate fermentation process for the purpose of purification [21].

## 5. Conclusions

The application of isolates from the *Alcaligenes* and *Bacillus* genera as means of purification of postfermentation broth is a promising method of removing organic acids.

However imperfect the procedures described in this work are and further investigation is necessary, such approach gives hope that the production of biodiesel may become soon a completely wasteless process. Furthermore, the removal of toxic by-products can lead to increased efficiency of the microbial synthesis of 1,3-PD and, in result, make the process more economically viable.

## Figures and Tables

**Table 1:** Compounds of postfermentative broth after 7 days of cultivation of the isolates.

Strain number	Utilization level						Time*
	Butyric acid	Acetic acid	Lactic acid	Ethanol	1,3-PD	Glycerol	
1	++	+++	+++	−	−	−	24 h
2	+	+++	+	−	−	+	NA
3	+	+++	+	−	−	+	NA
5	++	+++	+	−	−	−	NA
6	+	+	−	−	+	+	NA
7	−	+	−	−	−	+	NA
9	+	+++	++	−	−	+	NA
11	+	+++	+	−	−	−	NA
12	++	+++	++	−	−	−	96 h
15	++	+++	+++	−	−	−	120 h
17	−	+++	+	−	−	−	NA
19	+	+++	+++	−	−	−	96 h
21	+		++	−	−	−	NA
25	+	−	+	−	+	+	NA
29	−	−	++	−	+	+	NA

+++: complete utilization.

++: utilization of more than 50%.

+: insignificant utilization (<10%).

−: lack of utilization.

*Time until 90% organic acids utilization.

NA: not applicable.

**Table 2:** Identification on new isolates by amplification of 16S rRNA.

Strains number	Isolation source	Species	Homology to geneare
1	Fermented silage with manure	*Alcaligenes faecalis *	99%
5	Fermented silage with manure	*Alcaligenes faecalis *	98%
12	Fermented silage with manure	*Alcaligenes faecalis *	97%
15	Fermented silage with manure	*Alcaligenes faecalis *	98%
21	Fermented silage with manure	*Alcaligenes faecalis *	96%
3	Fermented chocolate	*Bacillus licheniformis *	98%
9	Fermented silage with manure	*Bacillus licheniformis *	97%
11	Fermented silage with manure	*Bacillus licheniformis *	96%
12	Fermented chocolate	*Bacillus licheniformis *	97%
